# Hemogram Findings in Cats from an Area Endemic for *Leishmania infantum* and Feline Immunodeficiency Virus Infections

**DOI:** 10.3390/vetsci9090508

**Published:** 2022-09-16

**Authors:** Marisa Masucci, Giulia Donato, Maria Flaminia Persichetti, Vito Priolo, Germano Castelli, Federica Bruno, Maria Grazia Pennisi

**Affiliations:** 1 Department of Veterinary Science, University of Messina, 98168 Messina, Italy; 2 National Reference Center for Leishmaniasis (C.Re.Na.L.), Istituto Zooprofilattico Sperimentale della Sicilia, 90129 Palermo, Italy

**Keywords:** feline, *Leishmania infantum*, FIV, complete blood count, anemia, monocytes, inflammation, thrombocytosis, South Italy

## Abstract

**Simple Summary:**

Some cats positive for *Leishmania infantum* (*Li*) show clinical signs and clinicopathological changes, including hemogram abnormalities. However, co-infections or co-morbidities are often detected in cats with leishmaniosis, and they can have a role in the clinical abnormalities observed. In feline *Leishmania infantum* infections and in clinical cases of feline leishmaniosis, a significant association with feline immunodeficiency virus (FIV) has been detected, but the role of this co-infection is controversial. The aims of this study were to retrospectively evaluate hemogram changes in cats from areas endemic for *Leishmania infantum* and feline immunodeficiency virus infections (Sicily and Calabria regions, Southern Italy) and to analyze the role of both pathogens in the abnormalities detected. A retrospective cross-sectional study was carried out on 496 cats. Hematological changes in cats positive for *Leishmania infantum* were found, particularly abnormalities compatible with chronic inflammation including moderate anemia, monocytosis, and morphologically activated monocytes. Moreover, some abnormalities, such as thrombocytosis, seem to arise when cats are co-infected with FIV. Feline leishmaniosis should be considered when some hemogram abnormalities compatible with chronic inflammation are detected.

**Abstract:**

In feline *Leishmania infantum* (*Li*) infection and in clinical cases of feline leishmaniosis, co-infection with feline immunodeficiency virus (FIV) has been reported. However, the role of the retroviral co-infection in the impairment of feline clinical health is still controversial. The aim of this study was to evaluate hemogram changes in cats from regions endemic for both *Li* and FIV infection. Four hundred and ninety-six cats tested for *Li* (EDTA blood polymerase chain reaction and immunofluorescence antibody test) and for FIV infection (enzyme-linked immune assay) were retrospectively evaluated. Hemogram results including blood smear morphological evaluation were statistically compared considering four infection patterns: *Li*+FIV+, *Li*+FIV−, *Li*−FIV+, and *Li*−FIV−. Significantly lower values of erythrocytes (*Li*+FIV−: *p* = 0.0248; *Li*−FIV+: *p* = 0.0392) and hemoglobin (*Li*+FIV: *p* = 0.0086; *Li*−FIV+: *p* = 0.0249) were found in both infections when compared to *Li*−FIV− cats, and severity of anemia was more frequently moderate in *Li*-positive cats (*p* = 0.0206) and severe in FIV infection (*p* = 0.024). *Li* infection was associated with monocytosis (*p* = 0.0013) and morphologically activated monocytes (*p* = 0.0209). Moreover, FIV infection was associated with the presence of inflammatory leukogram (*p* = 0.023), and an association between thrombocytosis and the co-infection was found (*p* = 0.0347). *Li* infection in cats induces hematological changes compatible with chronic inflammation, some of which are due to co-infection with FIV.

## 1. Introduction

Leishmaniosis is considered an emerging feline disease probably due to a higher level of cat veterinary care in endemic areas and more sensitive diagnostic techniques available than in the past [1]. Infection in cats is due to the same *Leishmania* species affecting dogs, and *Leishmania infantum* (*Li*) infection has been detected in cats from areas endemic for canine leishmaniosis, particularly in Mediterranean countries (Italy, Spain, Portugal, France, Greece, Turkey, Cyprus, Israel) [1,2,3,4,5,6,7,8], Latin America (Brazil) [1,6], and Asia (Iran) [1,6]. 

However, both *Li* infection and leishmaniosis are less frequent in cats compared to dogs [1,8]. It is likely that most of the infected cats do not develop a disease, possibly due to their innate or adaptive immune response [1,6]. Nevertheless, some cats show a variety of clinical signs [1,6,9] and clinicopathological changes [1,6]. Among the latter, complete blood count (CBC) abnormalities, such as anemia [9,10,11,12,13,14], leukocytosis [12,15,16] with neutrophilia [16], monocytosis [14], eosinophilia [14,15,17], leukopenia [9,10,18], and thrombocytopenia [9,10,19,20] have been reported. However, co-infections or co-morbidities are often detected in cats with leishmaniosis, and they can have a role in the clinical picture observed in cats positive to *Li* [1,4].

Most cats affected by leishmaniosis are suspected to have an impaired immune response due to concomitant infectious and immune-mediated diseases, or treatment with immunosuppressive drugs [1,6]. In feline *Li* infections and in clinical cases of feline leishmaniosis (FeL), a significant association with feline immunodeficiency virus (FIV) has been detected, but the role of this co-infection is controversial and still under study [1,2,6,14]. Hematological changes are among the most common abnormalities caused by FIV infection. They are caused by different pathomechanisms such as myelosuppression, immunosuppression, autoimmunity, and secondary infections. Anemia [21,22,23], leukopenia [22,23], neutropenia [21,22,24], lymphopenia [21,22], leukocytosis [22], neutrophilia [22], monocytosis [21,22], lymphocytosis [21], and thrombocytopenia [23] have been described. 

Few studies compared hematological abnormalities between *Li*-positive and -negative cats, and they did not find significant differences [5,8]. Spada et al. (2020) evaluated co-infections, but they found only one case of FIV co-infection in that study [5]. In a recent study reporting clinical signs and hematological abnormalities associated with *Li* and FIV co-infection, no CBC abnormalities were significantly associated with this co-infection [14]. 

The aims of this study were to retrospectively evaluate CBC values in cats from areas endemic for *Li* and FIV infections, such as Sicily and Calabria regions (Southern Italy) [2,25,26,27,28,29,30,31], and to analyze the role of both pathogens in the alterations detected. 

## 2. Materials and Methods

### 2.1. Power and Sample Size

Assuming an expected *Li* antibody prevalence of 14%, *Li* molecular prevalence of 4%, and FIV seroprevalence of 12.9% in Sicily and Calabria regions, a sample size of 499 (*Li* antibody prevalence), 159 (*Li* molecular prevalence), and 467 (FIV antibody prevalence) cats were required (99% CI; 4% level of precision) [29,30].

### 2.2. Study Sites, Cat Enrollment, and Clinical Classification and Sampling Procedures 

A retrospective cross-sectional study was carried out, analyzing results from three studies already published [28,29,30]. Cats were sampled between 2012 and 2019 at four veterinary clinics in Sicily (Ospedale Veterinario Universitario Didattico, Dipartimento di Scienze Veterinarie, Università di Messina, Messina; Ambulatorio Veterinario S. Lucia, Lipari, Messina) and Calabria (Clinica Veterinaria Camagna, Reggio Calabria; Ambulatorio Dr. Cardone, Gioia Tauro, Reggio Calabria) regions. Signalment, history, and physical examination findings were registered in a clinical form. According to physical examination, cats were classified as apparently healthy, affected by signs compatible with feline leishmaniosis [1], and affected by other signs. Clinical manifestations considered compatible with feline leishmaniosis were lymph node enlargement, skin and/or mucocutaneous lesions (mainly ulcerative or nodular), ocular lesions (mainly uveitis), oral lesions, weight loss, anorexia, lethargy, dehydration, pale mucous membranes, hepatomegaly, icterus, cachexia, fever, diarrhea, chronic nasal discharge, splenomegaly, polyuria/polydipsia, itching, dyspnea, wheezing, abortion, and hypothermia [1]. In most cases, cats included in this category showed multiple signs among those listed above. Signs and pathologies categorized as “other signs” were overweight, otitis, cystitis, and cough.

Cats were phlebotomized under physical restraint, and from each cat, about five milliliters of venous blood was collected. One milliliter was placed into a K_3_EDTA tube and used for CBC evaluation within two hours after collection or stored at +4 °C and used after a maximum of 24 h. The leftover K_3_EDTA blood was then stored at −20 °C until analyzed for detection of *Li* DNA or feline leukemia virus infection (FeLV) RNA. The remaining blood was used to make blood smears and to obtain serum after clotting in a dry tube and centrifugation. Each aliquot of serum was stored at −20 °C until analyzed for *Li* and FIV antibody detection, and FeLV p27 antigen. 

Informed consent was obtained from owners, and approval of the ethics committee from the author’s academic institution was obtained. 

### 2.3. Complete Blood Count 

Complete blood count was performed using a laser hematology analyzer ProCyte Dx (IDEXX laboratories, Westbrook, ME, USA). Reference intervals of CBC parameters are shown in Table 1. Blood smears were stained by May–Grünwald–Giemsa stain (Merck KgaA, Darmstadt, Germany) and evaluated microscopically at oil immersion ×1000 magnification [32].

Low platelet count as well as any “smart flag” message reported by the analyzer about leukocyte or platelet count (i.e., inability of the analyzer to make the count or inaccuracy of the analyzer count) were correspondingly confirmed or settled after microscopic examination of blood smears. Blood smears were also examined for cell morphological abnormalities and to exclude as thrombocytopenic samples where platelet clumps were observed. 

Cats were considered anemic when hemoglobin concentration was lower than 9.8 g/dL. Anemia severity was classified according to hematocrit (HCT) values as mild (HCT ≥ 20%), moderate (HCT 14–19%), or severe (HCT ≤ 13%) [33]. Anemia was considered regenerative or non-regenerative when reticulocyte count was higher or ≤ 50,000/µL, respectively. According to the mean cell volume (MCV), anemia was then classified into macrocytic (MCV > 53.1 fL), normocytic (MVC 35.9–53.1 fL), or microcytic (MCV < 35.9 fL) and according to the mean corpuscular hemoglobin concentration (MCHC) in normochromic (MCHC 28.1–35.8 g/dL) or hypochromic (MCHC < 28.1 mg/dL) cases.

Leukocyte alterations considered suggestive of inflammation included neutrophils left shift, toxic neutrophils, and reactive lymphocytes [34,35]. 

### 2.4. Diagnosis of L. infantum Infection

Anti-*Li* immunoglobulin G (IgG) antibodies (Ab) were tested by immunofluorescence antibody test (IFAT) using *Li* (strain MHOM/IT/80/IPT1) antigen slides produced by CReNaL (Centro di Referenza Nazionale per la Leishmaniosi, Palermo, Italy) following Office International des Epizooties (OIE) Terrestrial Manual protocol [36]. Fluoresceinated rabbit anti-cat IgG Ab (working anti-feline IgG (H+L)-FITC, Füller Laboratories, Fullertone, CA, USA) or fluoresceinated goat anti-cat IgG antibody (anti-cat IgG-FITC conjugate, SIGMA, Sant Louis, MI, USA) were used. The manufacturer’s protocol was followed, and the end-point titer of positive samples was determined preparing PBS serial twofold dilutions of serum. The cut off dilution value for positivity was set at 1:80 [37,38]. 

### 2.5. Polymerase Chain Reaction (PCR) 

DNA was extracted from blood EDTA using the PureLink Genomic DNA kit (Invitrogen, California, USA) in different steps. According to the manufacturer’s instructions, 200 µL of whole blood was employed for DNA extraction. At the end of the extraction procedure, DNA was eluted in 100 µL of PureLink genomic elution buffer and stored at −20 °C until used. The PCR test was targeted at the constant region in the minicircle Kinetoplast DNA (NCBI accession number AF291093). Real-time polymerase chain reaction of blood EDTA was developed by the CFX96 Real-time System (Bio-Rad Laboratories s.r.l.) using TaqMan Master Mix (Applied Biosystems). A multiplex PCR was optimized including an internal DNA control with specific probe and primer according to the VIC internal PC kit (Applied Biosystems). Real-time PCR was carried out in a final volume of 20 µL including a final concentration 1× of TaqMan Master Mix (Applied Biosystems); 0.3 µM of each primer (5′AAAATGGCATTTTCGGGCC-3′ and 5′-GGCGTTCTGCGAAAACCG-3′); 0.25 µM of the fluorogenic probe (5′-FAM-TGGGTGCAGAAATCCCGTTCA3′-BHQ1); and 50 ng of DNA, 1× Exo IPC Mix, and 1× Exo IPC DNA. The thermal cycle conditions consisted of 150 s initial incubation at 50 °C and 10 min of initial denaturation at 95 °C, followed by 40 cycles at 95 °C for 15 s and annealing–polymerization at 60 °C for 35 s according to the protocol of Castelli et al. [39]. Samples were amplified in a single 96-well plate, and in each one, a positive control containing genomic *L. infantum* DNA and a negative control without DNA were included. Each standard, sample, and negative control was analyzed in duplicate for each run. Cycle threshold (Ct) value was calculated for each sample by determining the point of the fluorescence value exceeding the threshold limit. The parasitic DNA load was defined in each examined sample by comparison of the data with a specific standard curve on the basis of the number of *Leishmania* per milliliter of extracted volume. Standard curves were prepared for both the *Leishmania* gene target and the internal PC (IPC Applied Biosystems). A stock solution of *L. infantum* DNA was obtained by extraction from 109 promastigotes/mL. Tenfold serial dilutions of the DNA stock solution were performed to obtain the six points of the curve spanning from 106 to 101 DNA equivalent cells. The standard curve, calculated by independent experiments, was linear over at least 6 log ranges of DNA concentration points with an average correlation coefficient of 0.988. The difference for each point of the curve was one log factor [39]. 

#### Diagnosis of Feline Retroviral Infections

Cats were tested for FIV and FeLV. Different diagnostic tests were used in the time interval considered for this retrospective cross-sectional study (2012–2019). In detail, anti-FIV antibodies were investigated with commercial kits (SNAP Combo Plus FeLV antigen and FIV antibody test, Idexx Laboratories, Westbrook, ME, USA (257 cats); Pet Check FIV anti- body test kit, IDEXX Laboratories, Westbrook, ME, USA (239 cats)). The FeLV positivity was assessed by a rapid ELISA test detecting p27 antigenemia (SNAP Combo Plus FeLV antigen and FIV antibody test, Idexx Laboratories, Westbrook, ME, USA (257 cats) or by blood real-time PCR (U3 region LTR-genesig^®^ Advanced kit, Rownhams, UK (239 cats)). All tests were performed according to the manufacturer’s protocol. 

### 2.6. Statistical Analysis

Statistical analysis was performed using GraphPad Prism version 7.0 for Windows (GraphPad Software, San Diego, CA, USA). Distribution of continuous variables was evaluated by D’Agostino–Pearson omnibus normality test, and descriptive statistics was performed for all the evaluated variables. 

Cats were classified according to four infection patterns: *Li* positive (IFAT and/or PCR) and FIV positive (*Li*+FIV+), *Li* positive and FIV negative (*Li*+FIV−), *Li* negative (IFAT and PCR) and FIV positive (*Li*−FIV+), and *Li* negative and FIV negative (*Li*−FIV−).

Fisher’s exact test was used to evaluate associations between the four infection patterns considered and signalment, history, clinical examination findings, CBC with blood smear morphological examination, degree of anemia, and its classification (regenerative/non regenerative, mycro/normo/macrocytic, hypo/normochromic). Parameters with 0 frequency were excluded from Fisher’s exact test. 

Kruskal–Wallis test and Dunn’s multiple comparisons test were used to compare CBC values among the four infection patterns observed. This was performed for all cats enrolled as well as for those with CBC alterations.

The Spearman’s rank correlation test was used to assess the correlation between antibody titer and hemogram values of cats with CBC abnormalities.

*p*-values < 0.05 were considered significant. 

## 3. Results

### 3.1. Cat Population Demographic and Clinical Data

Four hundred and ninety-six cats were enrolled: 319 from Calabria and 177 from Sicily regions. Signalment, history, and clinical data collected are shown in Table 2. Cats were aged between 5 and 228 months (median = 36 months, 25% percentile = 12 months, 75% percentile = 74 months).

Prevalence of males was significantly higher in *Li*−FIV+ than both *Li*+FIV− and *Li*−FIV− cats (Table 2 and Table 3). Moreover, prevalence of males was higher in *Li*+FIV+ than *Li*−FIV− animals (Table 2 and Table 3). Prevalence of adult cats was higher, when compared to young cats, in *Li*+FIV+ and *Li*−FIV+ than *Li*−FIV− (Table 2 and Table 3). Finally, prevalence of senior cats was higher compared to young cats in *Li*+FIV+, *Li*+FIV−, and *Li*−FIV+ than *Li*−FIV− (Table 2 and Table 3).

### 3.2. L. infantum and Retroviral Infections

Anti-*Li* antibodies were detected in 69 (13.9%) cats, *Li* DNA was found in 16 (3.2%) animals, and anti-FIV antibodies were detected in 50 (10.1%) cats. The four infection patterns were observed as follows: *Li*+FIV+ (*n* = 16), *Li*+FIV− (*n* = 61), *Li*−FIV+ (*n* = 34), and *Li*−FIV− (*n* = 385). All cats were FeLV negative. 

### 3.3. Complete Blood Count

Lower values of erythrocytes were detected in *Li*+FIV− and in *Li*−FIV+ cats compared to *Li*−FIV− cats (Table 4 and Table 5). Additionally, *Li*−FIV+ cats had lower values of hemoglobin than *Li*−FIV− cats (Table 4 and Table 5) and lower values of MCHC than *Li*+FIV+, *Li*+FIV−, and *Li*−FIV− cats (Table 4 and Table 5). When only anemic cats were considered, lower values of hemoglobin were detected in *Li*+FIV− cats (median = 7.05; 25–75° percentile = 6.05–8.2) than in *Li*−FIV− cats (median = 8.7; 25–75° percentile = 8–9.1) (Table 5).

Moderate anemia was more frequently observed when compared with mild anemia in *Li*+FIV− cats than *Li*−FIV− cats (Table 6 and Table 7). Moreover, severe anemia was more frequently found when compared to mild anemia in *Li*−FIV+ cats than *Li*−FIV− cats (Table 6 and Table 7). Finally, hypochromic anemia was more frequently found in *Li*−FIV+ (30.77%) than in *Li*−FIV− cats (7.53%), and macrocytic anemia was more frequently found in *Li*−FIV+ cats (36.36% and 50%, respectively) when compared to normocytic and microcytic cases than *Li*−FIV− cats (4.82% and 12.9%, respectively) (Table 7).

Data regarding reticulocyte hemoglobin content (Retic-Hb) were available for 113 hemograms (median = 14.6, range = 13–23.2; 25–75° percentile = 13.6–15.6). Normal Retic-Hb values were detected in 5 *Li*+FIV+ cats, 9 *Li*+FIV− cats, 7 *Li*−FIV+ cats, and in 91 *Li*−FIV− cats, and a low value was observed in only one cat. This domestic shorthair, male, young cat was apparently healthy and *Li*−FIV−, and neutropenia was the unique abnormality found in the CBC. 

Monocytosis and morphologically activated monocytes were more frequently observed in *Li*+FIV− cats compared to *Li*−FIV− cats (Table 8 and Table 9). Inflammatory leukogram was more frequently detected in *Li*−FIV+ cats than *Li*−FIV− cats (Table 8 and Table 9). 

Frequency of thrombocytosis was significantly higher in *Li*+FIV+ cats than *Li*−FIV− cats (Table 8 and Table 9).

No correlation was found between antibody titer against *Li* and CBC values. 

A statistical evaluation of the association between clinical signs and hematological alterations was not made because almost all the examined cats showed at least one clinical sign compatible with leishmaniosis (Table 10).

## 4. Discussion

In this retrospective cross-sectional study, a prevalence of 13.9% for *Li* antibody and 3.2% for *Li* DNA detection in blood samples was found, and 10.1% of cats had antibodies against FIV. Conversely, no cats were FeLV positive. These results confirm that Southern Italy, particularly Sicily and Calabria, is an endemic area for *Li* and FIV, while FeLV is sporadic.

According to the results of the present study, there was no significant association between anemia and infections with *Li* or FIV; however, significantly lower values of erythrocytes (*Li*+FIV− and *Li*−FIV+) and hemoglobin (*Li*−FIV+) were found in both infections when considering all results, and significantly lower values of hemoglobin were detected in *Li* infection (*Li*+FIV−) when only anemic cats were considered. Co-infection did not cause significantly lower values compared to the other patterns. However, the number of *Li*+FIV+ cats was small (16), and a higher number of co-infected cats should be analyzed. Severity of anemia was more frequently moderate in cats infected with *Li* and severe in the case of FIV infection. A considerable percentage of *Li*−FIV− cats had anemia (28.8%), and it was mostly a mild anemia (88.2%). However, only 21.3% of *Li*−FIV− cats were apparently healthy at physical examination and, unfortunately, we could not analyze data about other pathogens and pathologies potentially causing anemia in the studied cats. Additionally, FIV-infected cats had more frequently lower MCHC values and presented hypochromic and macrocytic anemia.

Mild-to-severe normocytic normochromic non-regenerative anemia is the most frequent hematological abnormality reported in clinical cases of feline leishmaniosis [1,6]. Anemia is also one of the most common laboratory findings in dogs with leishmaniosis with multiple and not fully known pathomechanisms [40,41,42,43,44,45,46]. In fact, blood loss, hemolysis, renal failure, chronic inflammation, and bone marrow alterations may be responsible for anemia in dogs with leishmaniosis. Erythroid hypoplasia [19,47,48,49]; erythroid dysplasia [50]; myelophthisis with infiltration of lymphocytes, plasma cells, and macrophages [19]; and erythrophagocytosis of erythroblasts [50] are the bone marrow alterations found in canine leishmaniosis. Interleukins produced by Th1 cells, such as IFN-γ, would play a role in the pathogenesis of anemia through inhibition of the earliest stages of erythroid differentiation and proliferation [51], alteration of iron homeostasis, induction of iron retention in macrophages, and consequent limitation of iron availability during erythropoiesis [49,51,52]. Moreover, the increased number of activated macrophages typical of leishmaniosis can promote erythrophagocytosis [53]. Changes in erythrocyte membrane fluidity, which may lead to their sequestration in the spleen, was also related to anemia in canine leishmaniosis [54]. Currently, there is a lack of data on the possible pathogenesis of anemia in feline leishmaniosis.

Both regenerative and non-regenerative anemia are commonly observed in FIV-infected cats because of a direct pathogenic effect of the virus. Multifactorial mechanisms have been proposed: ineffective hematopoiesis due to FIV infection of the bone marrow stromal cells that alters normal hematopoietic functions [55] and immunomediated destruction of erythrocytes due to an overactive immune response [56,57]. However, concomitant diseases often play an important role. This is the case of hemolytic anemia caused by hemotropic mycoplasmas [58,59]. Moreover, suppression of the immune system induced by FIV is associated with chronic opportunistic infections responsible for various hematological changes included normocytic normochromic non-regenerative anemia [23].

Most of the morphological alterations of erythrocytes were very rare and, when it was possible to use Fisher’s exact test, no significant differences were found according to the infection pattern.

We found that *Li* infection was associated with monocytosis and with the occurrence of morphologically activated monocytes. Monocytosis has rarely been reported in *Li*-infected cats [14]. Conversely, it is considered a finding consistent with leishmaniosis in dogs [46], and higher monocyte concentrations have been detected in dogs with positive splenic culture when compared with dogs with negative spleen culture [49], in dogs with leishmaniosis when compared to healthy dogs [60], and in oligosymptomatic *Li*-infected dogs when compared to non-infected dogs [47].

Monocytes play a crucial role in *Leishmania* infection, contributing to both innate immune defense and adaptative immune responses [47,61,62]. Moreover, monocytosis occurs in many conditions in cats, including acute and chronic inflammation and tissue destruction due to trauma-related injuries, suppuration, necrosis, pyogranulomatous inflammation, hemolysis, malignancy, and immune-mediated disorders [34].

In the present study, FIV infection was associated with the presence of inflammatory leukogram. FIV-induced immunosuppression could have facilitated a secondary or opportunistic infection, to which an appropriate inflammatory response was made [22]. Many FIV-infected cats, with concomitant or secondary infections, still have an appropriate hematological response and show leukocytosis, neutrophilia, and left shift in association with purulent or inflammatory processes [59]. Neutrophilia and monocytosis were common hematological abnormalities seen in cats with naturally occurring FIV infection in previous studies [14,21,22,63]. We could not evaluate the clinical stage of FIV-positive cats under study but, on the basis of the association of the retroviral infection with the occurrence of inflammatory leukograms, they were not in the end stage of disease.

We found an association between thrombocytosis and the co-infection by *Li* and FIV, although this infection pattern involved only 11 cats, and this abnormality affected a small number of cats (2/11). Thrombocytosis has never been described before in cats with *Li* or FIV infection and in the case of co-infection. Thrombocytosis has been found in dogs with *Li* infection, and it was assumed that it can be related to the chronic inflammatory process [44]. The so-called reactive thrombocytosis is the most common form of thrombocytosis in dogs and cats, and it is caused by a high level of inflammatory cytokines that stimulate thrombopoiesis [64]. Importantly, the two cases of thrombocytosis occurred in cats with an inflammatory leukogram. This supports the hypothesis of a reactive mechanism for thrombocytosis of cats coinfected with *Li* and FIV.

We evaluated for the first time the reticulocyte hemoglobin content in *Li*- and FIV-positive cats. The rationale for investigating this index was that chronic inflammation is associated with functional iron deficiency and can cause a non-regenerative normocytic normochromic anemia. Reticulocyte hemoglobin content is one of the reticulocyte indices considered as markers of iron deficiency and has been recently included in the hemogram variables investigated by veterinary laser hematology analyzers. As it was introduced in the analyzer used in this study in 2019, we were able to include this variable in a limited number of cats (113). Most of them (81%) were negative to both the investigated infections, and a low value was found in only one negative cat. As far as we know, only two studies investigated reticulocyte hemoglobin content in cats, and Keiner et al. (2020) found that sensitivity for iron-limited erythropoiesis was low [65,66]. Further clinical studies about cat reticulocyte indices are indeed needed.

As previously reported, a significant association between *Li* antibody positivity and old age was detected [1,6,31]. This finding can be explained by a long-lasting course of antibody positivity after cat seroconversion and a good life expectancy for antibody-positive cats. When these two latter conditions are met, in *Li*-endemic areas where no preventative measures are implemented, the number of seropositive cats can increase year by year, and prevalence will be higher according to the number of transmission seasons the cat passed.

Moreover, male sex [22,24,67] and adulthood or old age were found to be risk factors for FIV infection [21,22,67], as largely reported in the literature.

This study has some limitations, and they are related to the retrospective approach. In fact, this has prevented the possibility to better assess relationships between hematological changes and the clinical status of cats. In particular, the stage of the disease was not considered for FIV-positive cats. This could have been a bias, as most cats do not exhibit immunosuppressive or myelosuppressive effects for a long time [56], and hematological sequelae of co-infection possibly depend on the cat FIV stage. Moreover, apart from FeLV infection, other pathogens were not considered, as well as co-morbidities affecting the hemogram. Additionally, methods used for testing retroviral infections were inhomogeneous. However, all the different tests we used have high sensitivity and specificity and are widely used in relevant studies regarding feline pathogens [68,69,70]. Finally, despite the high prevalence found for *Li* and FIV, the infection pattern that included co-infected cats (*Li*+FIV+) consisted of a small number of subjects. A more homogeneous number of samples is required in each category to obtain a robust analysis of results.

## 5. Conclusions

In conclusion, *Li* infection in cats is associated with hematological changes; is compatible with a chronic inflammation; and includes moderate anemia, monocytosis, and morphologically activated monocytes. Some of them, such as thrombocytosis, arise in the case of co-infection with FIV and should be further investigated. The hemogram obtained with a routine test such as CBC can therefore provide clinicians with data supporting a suspicion of feline leishmaniosis when alterations suggestive of chronic inflammation are found.

## Figures and Tables

**Table 1:** IDEXX ProCyte Dx reference intervals of complete blood count (CBC) parameters considered for statistical evaluation.

Parameters	Unit of Measure	Reference Interval
Red blood cells	M/µL	6.54–12.2
Hematocrit	%	30.3–52.3
Hemoglobin	g/dL	9.8–16.2
MCV	fL	35.9–53.1
MCHC	g/dL	28.1–35.8
Reticulocytes	K/µL	3–50
Retic-Hb	pg	13.2–20.8
White blood cells	K/µL	2.87–17.02
Neutrophils	K/µL	2.3–10.29
Lymphocytes	K/µL	0.92–6.88
Monocytes	K/µL	0.05–0.67
Eosinophils	K/µL	0.17–1.57
Basophils	K/µL	0.01–0.26
Platelets	K/µL	151–600

MCV = mean cell volume; MCHC = mean corpuscular hemoglobin concentration; Retic-Hb = reticulocyte hemoglobin content.

**Table 2:** Data from signalment, history, and clinical examination (*n* (%)) of total enrolled cats (total) and of the cats classified according to their infection pattern (*Li*+FIV+; *Li*+FIV−; *Li*−FIV+; *Li*−FIV−).

Signalment, History, and Clinical Data	Total	*Li*+FIV+	*Li*+FIV−	*Li*−FIV+	*Li*−FIV−
**Region**					
Calabria	319 (64.3)	8/16 (50)	42/61 (68.8)	25/34 (73.5)	244/385 (63.4)
Sicily	177 (35.7)	8/16 (50)	19/61 (31.1)	9/34 (26.5)	141/385 (36.6)
**Sex**					
Male	212 (42.7)	11/16 * (68.7)	25/61 * (41)	22/34 * (64.7)	154/385 * (40)
Female	284 (57.3)	5/16 (31.2)	36/61 (59)	12/34 (35.3)	231/385 (60)
**Breed**					
DSH and mixed breed	473 (95.4)	16/16 (100)	58/61 (95.1)	34/34 (100)	365/385 (94.8)
Pure breed	23 (4.6)	0/16	3/61 (4.9)	0/34	20/385 (5.2)
**Age group**					
Young	230 (46.4)	2/16 (12.5)	23/61 (37.7)	6/34 (17.6)	199/380 (52.4)
Adult	182 (36.7)	8/16 * (50)	23/61 (37.7)	16/34 * (47.1)	135/380 * (35.5)
Senior	79 (15.9)	6/16 * (37.5)	15/61* (24.6)	12/34 * (35.3)	46/380 * (12.1)
Not known	5 (1)				
**Lifestyle**					
Indoor	166 (33.5)	3/16 (18.75)	21/60 (35)	9/34 (26.5)	133/384 (34.6)
Outdoor	328 (66.1)	13/16 (81.25)	39/60 (65)	25/34 (73.5)	251/384 (65.4)
Not known	2 (0.4)				
**Clinical status**					
Apparently healthy	94 (18.9)	1/16 (6.25)	8/61 (13.1)	3/34 (8.8)	82/385 (21.3)
Signs compatible with FeL	367 (74)	15/16 (93.7)	51/61 (83.6)	31/34 (91.2)	270/385 (70.1)
Other signs	35 (7.1)	0/16	2/61 (3.3)	0/34	33/385 (8.6)

DSH: domestic shorthair; young ≤ 24 months, adult 25–96 months, senior > 96 months; FeL: feline leishmaniosis; * = significant difference.

**Table 3:** *p*-value, odds ratio (OR), and 95% confidence interval (CI) for the significant differences concerning signalment, history, and clinical examination.

Signalment, History, and Clinical Data	*p*		OR	95%CI
**Sex**				
Male	*Li*−FIV+ vs. *Li*+FIV−	0.0333	2.64	1.13–6.11
Female	*Li*−FIV+ vs. *Li*−FIV−	0.0063	2.75	1.33–5.60
	*Li*+FIV+ vs. *Li*−FIV−	0.0350	3.30	1.11–8.67
**Age group**				
Young	*Li*+FIV+ vs. *Li*−FIV− °	0.0190	5.90	1.42–27.83
Adult	*Li*−FIV+ vs. *Li*−FIV− °	0.0036	3.93	1.53–9.92
Senior	*Li*+FIV+ vs. *Li*−FIV− ^#^	0.0012	12.98	3.08–63.97
	*Li*+FIV− vs. *Li*−FIV− ^#^	0.0095	2.82	1.34–5.87
	*Li*−FIV+ vs. *Li-FIV−*^#^	<0.0001	8.65	3.09–23.97

° adult vs. young; ^#^ senior vs. young.

**Table 4:** Median (minimum–maximum) (25° percentile; 75° percentile) of complete blood count (CBC) values of cats classified according to their infection patterns considered (*Li*+FIV+; *Li*+FIV−; *Li*−FIV+; *Li*−FIV−).

CBC Parameters	*Li*+FIV+	*Li*+FIV−	*Li*−FIV+	*Li*−FIV−
Red blood cells (M/µL)	7.92	7.39 *	7.17 *	8.31 *
	(4–9.75)	(3.78–11.81)	(2.03–17.41)	(2.42–16.78)
	[5.49–9.08]	[5.92–9.02]	[6.01–8.9]	[7.11–9.38]
Hematocrit (%)	30.45	31.4	32.85	34.4
	(16–41.1)	(14.8–49.7)	(9.8–73.4)	(10.1–82.4)
	[22.95–36.5]	[26.95–36.75]	[25.78–38.68]	[29.15–38.6]
Hemoglobin (g/dL)	10.65	10.5	10.1 *	11.3 *
	(6.3–12.6)	(4.8–16.3)	(3.4–22.4)	(3.4–22.8)
	[7.65–12.38]	[8.2–12.1]	[8.5–11.35]	[9.5–12.85]
MCV (fL)	39.8	40.8	42.6	40.8
	(34–58)	(32.9–61.4)	(31–61.6)	(24.9–64.1)
	[38.05–42.03]	[39.1–42.8]	[37.08–49.93]	[37.8–43.75]
MCHC (g/dL)	34 *	33.9 *	30.65 *	33.4 *
	(30.2–47.8)	(26.5–40.2)	(26–42.3)	(14.1–68.5)
	[32.23–36.55]	[31.6–34.85]	[28.85–34.23]	[31.1–34.9]
White blood cells (K/µL)	12.48	11.87	13.72	11.77
	(4.24–25.9)	(3.4–56.85)	(7.19–32.01)	(0.1–74.96)
	[8.13–16.31]	[8.61–16.46]	[9.95–19.58]	[8.42–16.26]
Neutrophils (K/µL)	7.91	6.57	8.34	6.85
	(2.07–19.86)	(1.42–41.1)	(2.98–27.85)	(0.02–70.92)
	[4.86–11.4]	[4.3–10.43]	[5.89–12]	[4.51–10.88]
Lymphocytes (K/µL)	2.68	3.24	3.11	2.96
	(0.72–8.5)	(0.76–11.75)	(0.14–13.14)	(0.05–14.12)
	[1.85–4.91]	[2.43–4.29]	[2.12–4.24]	[2.15–4.32]
Monocytes (K/µL)	0.52	0.46	0.49	0.37
	(0.12–1.07)	(0.06–5.39)	(0–1.68)	(0–52)
	[0.28–0.87]	[0.28–0.81]	[0.34–0.75]	[0.25–0.56]
Eosinophils (K/µL)	0.48	0.69	0.59	0.64
	(0–0.95)	(0–4.15)	(0–2)	(0–7.47)
	[0.16–0.58]	[0.37–0.93]	[0.2–0.9]	[0.36–0.96]
Basophils (K/µL)	0.03	0.06	0.05	0.05
	(0–0.07)	(0–0.31)	(0–0.18)	(0–0.63)
	[0.01–0.04]	[0.04–0.1]	[0.02–0.08]	[0.03–0.09]
Platelets (K/µL)	367	297	336.5	276
	(151–641)	(3–670)	(9–1051)	(0–844)
	[224–525]	[212–407.5]	[227–496.3]	[172–376]

* = significant difference; MCV = mean cell volume; MCHC = mean corpuscular hemoglobin concentration.

**Table 5:** *p*-values for the significant differences concerning CBC values.

CBC Parameters	*p*
Red blood cells (M/ µL)	*Li*+FIV− vs. *Li*−FIV− = 0.0248 *Li*−FIV+ vs. *Li-FIV−* = 0.0392
Hemoglobin (g/dL)	*Li*−FIV+ vs. *Li*−FIV− = 0.0249 *Li*+FIV− vs. *Li*−FIV− = 0.0086 °
MCHC (g/dL)	*Li*−FIV+ vs. *Li*+FIV+ = 0.0486 *Li*−FIV+ vs. *Li*+FIV− = 0.0418 *Li*−FIV+ vs. *Li*−FIV− = 0.0168

° *p*-value obtained when only anemic cats were considered; MCHC = mean corpuscular hemoglobin concentration.

**Table 6:** Prevalence of different types of anemia (*n* (%)) of cats classified according to their infection pattern (*Li*+FIV+; *Li*+FIV−; *Li*−FIV+; *Li*−FIV−).

Type of Anemia	*Li*+FIV+	*Li*+FIV−	*Li*−FIV+	*Li*−FIV−
Mild	4/6 (66.7)	14/20 (70)	11/15 (73.3)	97/110 (88.2)
Moderate	2/6 (33.3)	6/20 * (30)	1/15 (6.7)	10/110 * (9.1)
Severe	0/6	0/20	3/15 * (20)	3/110 * (2.7)
Non-regenerative	6/6 (100)	15/20 (75)	12/15 (80)	102/110 (92.7)
Regenerative	0/6	5/20 (25)	3/15 (20)	6/110 (5.4)
Missing data	0/6	0	0/15	2/110 (1.8)
Microcytic hypochromic	0/6	0/20	0/15	0/110
Microcytic normochromic	0/6	2/20 (10)	3/15 (20)	17/110 (15.4)
Normocytic hypochromic	0/6	2/20 (10)	1/15 (6.7)	4/110 (3.6)
Normocytic normochromic	4/6 (66.7)	14/20 (70)	5/15 (33.3)	68/110 (61.8)
Macrocytic hypochromic	0/6	0/20	3/15 (20)	3/110 (2.7)
Macrocytic normochromic	0/6	0/20	1/15 (6.7)	1/110 (0.9)
Microcytic °	1/6 (16.7)	1/20 (5)	1/15 (6.7)	10/110 (9.1)
Normocytic °	1/6 (16.7)	1/20 (5)	1/15 (6.7)	7/110 (6.4)

* Significant difference; ° incomplete classification of sample because of mean corpuscular hemoglobin concentration value over upper reference level.

**Table 7:** *p*-value, odds ratio (OR), and 95% confidence interval (CI) for the significant differences concerning the types of anemia.

Type of Anemia	*p*		OR	95%CI
Mild	*Li*+FIV− vs. *Li*−FIV− °	0.0206	4.16	1.21–13.65
Moderate				
Severe	*Li*−FIV+ vs. *Li*−FIV− ^#^	0.0240	8.82	1.82–40.03
Hypochromic	*Li*−FIV+ vs. *Li*−FIV−	0.0282	5.46	1.52–19.48
Normochromic				
Microcytic	*Li*−FIV+ vs. *Li*−FIV− ^	0.0058	11.29	2.68–43.78
Normocytic				
Macrocytic	*Li*−FIV+ vs. *Li*−FIV− ^&^	0.0410	6.75	1.38–38.52

° mild vs. moderate; ^#^ mild vs. severe; ^ macrocytic vs. normocytic; ^&^ macrocytic vs. microcytic.

**Table 8:** Prevalence of complete blood count (CBC) abnormalities (*n* (%)) in total enrolled cats (total) and of cats classified according to their infection pattern (*Li*+FIV+; *Li*+FIV−; *Li*−FIV+; *Li*−FIV−).

CBC Abnormalities	Total ^§^	*Li*+FIV+ ^#^	*Li*+FIV− ^#^	*Li*−FIV+ ^#^	*Li*−FIV− ^#^
Anemia	151/496 (30.4)	6/16 (37.5)	20/60 (33.3)	15/33 (45.4)	110/382 (28.8)
Erythrocytosis	5/496 (1)	0/10	1/41 (2.4)	1/19 (5.3)	3/275 (1.1)
Leukopenia	3/493 (0.6)	0/13	0/47	0/23	3/298 (1)
Leukocytosis	112/493 (22.7)	3/16 (18.7)	14/61 (22.9)	11/34 (32.3)	84/379 (22.2)
Neutropenia	26/492 (5.3)	1/10 (10)	3/45 (6.7)	0/22	22/280 (7.9)
Neutrophilia	135/492 (27.4)	6/15 (40)	16/58 (27.6)	12/34 (35.3)	101/359 (28.1)
Lymphopenia	22/492 (4.5)	2/16 (12.5)	1/60 (1.7)	2/31 (6.4)	17/373 (4.6)
Lymphocytosis	12/492 (2.4)	0/14	1/60 (1.7)	3/32 (9.4)	8/364 (2.2)
Monocytosis	94/492 (19.1)	5/16 (31.2)	21/61 * (34.4)	9/32 (28.1)	59/368 * (16)
Eosinopenia	53/489 (10.8)	4/15 (26.7)	6/54 (11.1)	6/32 (18.7)	37/345 (10.7)
Eosinophilia	43/489 (8.8)	0/11	7/55 (12.7)	1/27 (3.7)	35/343 (10.2)
Basophilia	11/489 (2.2)	0/14	1/59 (1.7)	0/30	10/336 (3)
Thrombocytopenia	34/471 (7.2)	0/9	4/53 (7.5)	1/29 (3.4)	29/363 (8)
Thrombocytosis	17/471 (3.6)	2/11 * (18.2)	4/53 (7.5)	3/31 (9.7)	8/342 * (2.3)
Polychromasia	11/411 (2.7)	0/8	2/48 (4.2)	2/29 (6.9)	7/326 (2.1)
Poykilocytosis	6/411 (1.4)	0/8	0/48	0/29	6/326 (1.8)
Acanthocytes	2/411 (0.5)	0/8	1/48 (2.1)	0/29	1/326 (0.3)
Keratocytes	2/411 (0.5)	0/8	0/48	0/29	2/326 (0.6)
Spherocytes	4/411 (1)	0/8	0/48	0/29	4/322 (1.2)
Ghost cells	3/411 (0.7)	0/8	0/48	0/29	3/326 (0.9)
Knizocytes	1/411 (0.2)	0/8	0/48	0/29	1/326 (0.3)
Ovalocytes	2/411 (0.5)	0/8	0/48	0/29	2/326 (0.6)
Dacryocytes	3/411 (0.7)	0/8	0/48	0/29	3/326 (0.9)
Schistocytes	1/411 (0.2)	0/8	0/48	0/29	1/326 (0.3)
Heinz bodies	1/411 (0.2)	0/8	0/48	0/29	1/326 (0.3)
Howell–Jolly bodies	24/411 (5.8)	0/8	3/48 (6.2)	2/29 (6.9)	19/326 (5.8)
Nucleated erythrocytes	15/411 (3.6)	0/8	3/48 (6.2)	3/29 (10.3)	9/326 (2.8)
Morphologically activated monocytes	18/411 (4.4)	1/7 (14.3)	5/47 * (10.6)	3/29 (10.3)	9/328 * (2.7)
Inflammatory leukogram	78/411 (19)	2/7 (28.6)	10/47 (21.3)	12/29 * (41.4)	54/328 * (16.5)

* = significant difference; ^§^ prevalence of individual anomalies was evaluated on different numbers of cats because some parameters have not been evaluated in some samples for technical reasons; ^#^ the denominator refers to the sum of the animals that presented the alteration and of that had normal values.

**Table 9:** *p*-value, odds ratio (OR), and 95% confidence interval (CI) for the significant differences concerning the CBC abnormalities.

CBC Abnormalities	*p*		OR	95%CI
Monocytosis	*Li*+FIV− vs. *Li*−FIV−	0.0013	2.75	1.51–4.91
Thrombocytosis	*Li*+FIV+ vs. *Li*−FIV−	0.03470	9.28	1.75–40.41
Morphologically activated monocytes	*Li*+FIV− vs. *Li*−FIV−	0.0209	4.22	1.51–12.90
Inflammatory leukogram	*Li*−FIV+ vs. *Li*−FIV−	0.0230	3.58	1.58–8.03

**Table 10:** Description of hematological abnormalities observed in total enrolled cats with number of cats (*n*) presenting each of them. The prevalence of each abnormality is reported (*n* (%)) in cats classified according to their infection pattern (*Li*+FIV+; *Li*+FIV−; *Li*−FIV+; *Li*−FIV−) and clinical status (apparently healthy, signs compatible with FeL, different/other signs).

Hematological Abnormalities and Cat Clinical Classification	*Li*+FIV+	*Li*+FIV−	*Li*−FIV+	*Li*−FIV−
**Anemia** (151)				
Apparently healthy	1/6 (16.7)	2/20 (10)	0/15	18/110 (16.4)
Signs compatible with FeL	5/6 (83.3)	18/20 (90)	15/15 (100)	85/110 (77.3)
Other signs	0/6	0/20	0/15	7/110 (6.4)
**Erythrocytosis** (5)				
Apparently healthy	0/0	1/1(100)	0/0	0/4
Signs compatible with FeL	0/0	0/1	1/1(100)	3/3(100)
Other signs	0/0	0/1	0/0	0/3
**Neutropenia** (26)				
Apparently healthy	1/1 (100)	0/3	0/0	5/22(22.7)
Signs compatible with FeL	0/1	3/3(100)	0/0	15/22(68.2)
Other signs	0/1	0/3	0/0	2/22 (9.1)
**Neutrophilia** (135)				
Apparently healthy	0/6	3/16 (18.75)	0/12	14/101 (13.9)
Signs compatible with FeL	6/6 (100)	13/16 (81.25)	12/12 (100)	83/101 (82.2)
Other signs	0/6	0/16	0/12	4/101 (4)
**Lymphopenia** (22)				
Apparently healthy	0/2	0/1	0/2	2/17 (11.8)
Signs compatible with FeL	2/2 (100)	1/1 (100)	2/2 (100)	14/17 (82.3)
Other signs	0/2	0/1	0/2	1/17 (5.9)
**Lymphocytosis** (12)				
Apparently healthy	0/0	1/1 (100)	0/3	2/8 (25)
Signs compatible with FeL	0/0	0/1	3/3 (100)	6/8 (75)
Other signs	0/0	0/1	0/0	0/0
**Monocytosis** (94)				
Apparently healthy	0/5	3/21 (14.3)	0/9	13/59 (22)
Signs compatible with FeL	5/5 (100)	18/21 (85.7)	9/9 (100)	42/59 (71.2)
Other signs	0/5	0/21	0/9	4/59 (6.8)
**Eosinopenia** (53)				
Apparently healthy	1/4 (25)	0/6	0/6	3/37 (8.1)
Signs compatible with FeL	3/4 (75)	6/6 (100)	6/6 (100)	32/37 (86.5)
Other signs	0/4	0/6	0/6	2/37 (5.4)
**Eosinophilia** (43)				
Apparently healthy	0/0	1/7 (14.3)	0/1	9/35 (25.7)
Signs compatible with FeL	0/0	6/7 (85.7)	1/1 (100)	25/35 (71.4)
Other signs	0/0	0/7	0/1	1/35 (2.9)
**Basophilia** (11)				
Apparently healthy	0/0	1/1 (100)	0/0	1/10 (10)
Signs compatible with FeL	0/0	0/1	0/0	9/10 (90)
Other signs	0/0	0/1	0/0	0/0
**Thrombocytopenia** (34)				
Apparently healthy	0/0	0/4	1/1 (100)	9/29 (31)
Signs compatible with FeL	0/0	4/4 (100)	0/1	20/29 (69)
Other signs	0/0	0/4	0/1	0/29
**Thrombocytosis** (17)				
Apparently healthy	0/2	2/4 (50)	0/3	3/8 (37.5)
Signs compatible with FeL	2/2 (100)	2/4 (50)	3/3 (100)	5/8 (62.5)
Other signs	0/2	0/4	0/3	0/8
**Howell–Jolly bodies** (24)				
Apparently healthy	0/0	0/2	0/3	1/19 (5.3)
Signs compatible with FeL	0/0	2/2 (100)	3/3 (100)	17/19 (89.5)
Other signs	0/0	0/0	0/0	1/19 (5.3)
**Nucleated erythrocytes** (15)				
Apparently healthy	0/0	0/3	1/3 (33.3)	0/9
Signs compatible with FeL	0/0	3/3 (100)	2/3 (66.7)	7/9 (77.8)
Other signs	0/0	0/3	0/3	2/9 (22.2)
**Morphologically activated monocytes** (18)				
Apparently healthy	0/1	2/5 (40)	1/3 (33.3)	2/9 (22.2)
Signs compatible with FeL	1/1 (100)	3/5 (60)	2/3 (67.7)	6/9 (66.7)
Other signs	0/1	0/5	0/3	1/9 (11.1)
**Inflammatory leukogram** (78)				
Apparently healthy	0/2	1/10 (10)	1/12 (8.3)	7/54 (13)
Signs compatible with FeL	2/2 (100)	9/10 (90)	11/12 (91.7)	44/54 (81.5)
Other signs	0/2	0/10	0/12	3/54 (5.5)

FeL= feline leishmaniosis.

## Data Availability

The data set analyzed for the current study is available from the corresponding author upon reasonable request.
